# Dietary high-polyphenols extra-virgin olive oil is effective in reducing cholesterol content in eggs

**DOI:** 10.1186/s12944-015-0001-x

**Published:** 2015-02-07

**Authors:** Vito Laudadio, Edmondo Ceci, Nunzia M B Lastella, Vincenzo Tufarelli

**Affiliations:** Department of Emergency and Organ Transplantation (DETO), Section of Veterinary Science and Animal Production, University of Study of Bari ‘Aldo Moro’, Valenzano 70010 Bari, Italy; Department of Veterinary Medicine, University of Bari ‘Aldo Moro’, Valenzano 70010 Bari, Italy

**Keywords:** Extra-virgin olive oil, Cholesterol, Egg quality

## Abstract

**Background:**

Extra-virgin olive oil (EVOO) represents an important food in Mediterranean diet due to its favorable effects on human and animal health derived from the consumption of polyphenols. We studied the effects of dietary EVOO differing in polyphenols levels on egg quality.

**Methods:**

A total of 150 laying hens were allotted into three groups over 10 weeks of the experimental period. The three diets were based on wheat-soybean meal with added oils at 2.5%. Hens were fed the following diets: (1) commercial diet containing sunflower oil (Control), (2) diet EVOO from *Cima di Bitonto* variety (low-polyphenols content; Low-P), and (3) diet EVOO from *Coratina* variety (high-polyphenols content; High-P). The performance of the hen, the qualitative traits of eggs, and the fatty acid composition and cholesterol content of egg-yolk were measured.

**Results:**

None of the egg productive parameters studied were influenced by dietary treatment, except for yolk color score that was enhanced in hens fed the both EVOO diets (*P* < 0.05). Feeding high-polyphenols EVOO reduced serum cholesterol level in hens (*P* < 0.01) and egg-yolk cholesterol levels (as per egg; *P* < 0.05). The dietary supplementation of high-polyphenols EVOO raised the polyunsaturated fatty acids (PUFAs) composition and increased the content of oleic and linolenic acids in egg-yolk. Moreover, the atherogenic index in egg-yolk decreased linearly in accordance with increasing levels of dietary polyphenols (*P* < 0.01).

**Conclusion:**

In conclusion, a diet for hens consisting of high-polyphenols level from extra-virgin olive oil can improve the fatty acid quality of egg-yolk while lowering the egg-yolk cholesterol level, which could be a beneficial functional food for human health.

## Background

The Mediterranean diet, in which olive oil is the major fat component, has been associated with a lower incidence of coronary heart disease [1]. Extra-virgin olive oil (EVOO) is rich in phenolic compounds which have been shown to delay in vitro metal-induced and radical-dependent low density lipoprotein oxidation [2].

The phenolic content of olive oil depends on a number of factors, but it is mainly dependent on the olive variety as well as oil production and storage [3]. The EVOO, obtained from the fruit of the olive tree solely by mechanical or other physical means under conditions that do not lead to oil alteration, has shown anti-inflammatory, immunomodulatory, antiproliferative and anti-apoptotic effects [4,5]. Traditionally, these health-protective effects have been ascribed to its high polyunsaturated fatty acids (PUFAs) content [6], although, nowadays, it is clear that many of the beneficial effects of EVOO intake are due to its minor highly bioactive components. Phenolic compounds are thought to be related to these beneficial effects receiving the most attention [7]. Oleuropein derivatives, especially hydroxytyrosol, have been shown to have protective effects against markers associated with the atherogenic process [8,9]. Scientific evidence supports the potential use of nutraceuticals focused in polyphenols constituents as agents capable to prevent or accelerate healing of cardiovascular disease [10].

Eggs have been considered as a principle food item for human consumption over the history as they provide most of the nutrition as suggested by the recommended daily allowance. The variability in the quality and nutritional values of eggs has a significant impact on consumers’ health [11,12]. Although eggs are an important source of nutrients due to their high quality protein and variety of vitamins and minerals, it is often recommended that people restrict their consumption since egg yolk contains nearly 30% lipids and high cholesterol content [13]. Egg consumption is also believed to raise the risk of cardiovascular disease by increasing blood cholesterol levels in hyper-responders and diabetics [14,15] and those groups, as well as persons with elevated LDL-cholesterol levels and heart disease, should limit their dietary cholesterol intake. Dietary fats are known to influence cell membrane, tissues and egg-yolk lipid composition and plasma lipoprotein concentrations depending on their constructive fatty acid content. Due to the health benefits associated with the consumption of phenolic compounds, much research is necessary to enrich different foods of animal origin, such as eggs [16]. For this reason, several studies had been conducted to raise PUFA content in eggs by using dietary fat sources, such as natural oil containing PUFA [17]. Antioxidants of a kind of those natural polyphenols have been used as dietary supplementation [18].

In this way, the role of EVOO-derived bioactive polyphenols on egg quality has never been investigated. Therefore, the best of our knowledge and based on these considerations, the present study was aimed to evaluate the effects of dietary supplementation with EVOO from olive cultivars differing in polyphenols levels on egg quality and egg yolk cholesterol.

## Results and discussion

No literature was located on the effects dietary supplementation with EVOO from olive cultivars differing in polyphenols levels on laying hens productive performance and egg quality; thus, this subject should be considered as a new investigation. In Table 1 are reported the effects of diet including EVOO on the egg production and weight as well as egg qualitative traits of laying hens. The egg-laying rate (%) were statistically similar (P = 0.151) for all hens on experimental diets with a range of 92.1 in hens on control diet and 92.7 in hens on high-polyphenols diet, indicating uniformity in the laying pattern and quantity of egg laid by the hens fed EVOO as dietary lipid source. The egg weight was also similar (P = 0.062) among experimental hens, with a range 62.8 and 63.5 g for hens fed control or EVOO diet respectively, indicating that the inclusion of the alternative lipid source supported that this egg trait as also previously found by Celebi et al. [19]. The average weight of the eggs conformed and compared positively with values reported for layers in available literatures [20,21].

**Table 1:** **Effect of different dietary extra-virgin olive oil on egg productive and qualitative parameters**

**Item**	**Diet** ^**1**^				
	**Control**	**Low-P**	**High-P**	**SEM**	***P*** **-value**
Egg-laying rate, %	92.1	92.4	92.7	0.31	0.151
Egg weight, g	62.8	63.1	63.5	0.29	0.062
Egg mass, g/hen per day	57.8	58.3	58.8	0.19	0.051
FCR, g feed/g egg	1.99	1.98	1.96	0.07	0.055
Haugh unit	88.01	89. 21	89.41	0.45	0.088
Shell thickness (mm × 10^−2^)	0.33	0.32	0.33	<0.01	0.436
Shell strength (kg/cm^2^)	1.51	1.55	1.57	0.04	0.098
Broken + shell-less eggs, %	0.15	0.16	0.17	<0.01	0.312
Dirty eggs, %	0.40	0.38	0.39	<0.01	0.234
Egg components, %					
Yolk	24.5	25.1	25.3	0.17	0.051
Albumen	64.0	63.7	63.7	0.21	0.063
Shell	11.5	11.2	11.0	0.09	0.201
Yolk color score	11.01	12.52	12.63	0.23	0.039

^1^Diets consisted of: Control commercial diet containing sunflower oil; Low-P, low-polyphenols extra-virgin olive oil from *Cima di Bitonto* cultivar; High-P, high-polyphenols extra-virgin olive oil from *Coratina* cultivar.

Dietary treatment did not negatively influence any trait related to egg or shell quality (Table 1). The overall values obtained in the present study are quite acceptable for optimal egg quality for this age of hens (28 to 38 wk) in the medium-phase of production. The shell thickness and strength also had similar mean values (P = 0.436) among dietary treatments for each parameter, indicating a similar relative density for the eggs. The best index of internal egg quality is the Haugh unit, which is calculated by measuring the height of the inner thick albumen and the egg weight. As it is well known, a reduced Haugh unit indicates deterioration of egg freshness. In the present study, the Haugh unit values were similar (P = 0.088) for the eggs laid by the experimental laying birds. This result is consistent with previous studies where the Haugh unit on the day of lay was not affected by diet supplemented with sunflower or extra-virgin olive oil as the fatty acid and antioxidant source [19]. The egg yolk colour score was increased when both variety of EVOO were included into the diet compared with the control group fed sunflower oil (P = 0.039). The influence of EVOO on yolk color observed in our study may be related to the quantity of natural pigments contained in the alternative feed ingredient. In addition to affecting the yolk colour, phenol compounds play a key role as antioxidants in the healthy development of chick embryos, assuring a stronger immune response [22]. Nevertheless, it is clear that the dietary variation in polyphenols is not the only factor that produces individual differences: an important role of breed, age and season was reported [23].

Serum and egg-yolk total cholesterol decreased linearly for hens given diets supplemented with EVOO (Table 2). The concentrations of total cholesterol in the egg-yolk were within or lower the ranges reported in laying hens [24-26]. Feeding high-polyphenols may increase the oxidative stability and provide a source of compounds that are useful for human nutrition and health. High-polyphenols EVOO supplementation in hens’ diet markedly decreased (P < 0.01) serum and yolk total cholesterol levels. In particular, Low-P and High-P treatments reduced cholesterol by 6.76 and 10.68% (on a per g of yolk basis), respectively, whereas on a per total yolk basis, Low-P and High-P reduced cholesterol by 4.04 and 6.74%, respectively.

**Table 2:** **Effect of different dietary extra-virgin olive oil on serum and egg-yolk cholesterol levels and egg-yolk fatty acid profile**

**Item**	**Diet** ^**1**^				
	**Control**	**Low-P**	**High-P**	**SEM**	***P*** **-value**
**Serum**					
Cholesterol, mg/dl	124.5	100.2	97.6	6.86	0.004
**Yolk**					
Cholesterol, mg/g yolk	13.01	12.13	11.62	0.332	0.007
Cholesterol, mg total yolk	200.2	192.1	186.7	0.703	0.029
**Yolk fatty acid, % on total FA**					
C14:0 Myristic	2.02	1.74	0.63	0.011	0.032
C14:1 n-5 Myristoleic	1.11	1.55	0.47	0.013	0.010
C16:0 Palmitic	39.32	32.97	34.49	0.071	0.005
C16:1 n-7 Palmitoleic	2.01	3.02	2.11	0.052	0.052
C18:0 Stearic	15.06	16.77	16.51	0.045	0.059
C18:1 Oleic	27.22	29.66	29.32	0.062	0.011
C18:2 Linoleic	12.70	12.22	15.39	0.036	0.017
C18:3 Linolenic	0.56	1.07	1.08	0.010	0.010
Σ SFA	56.40	52.48	51.63	0.141	0.025
Σ MUFA	30.34	34.23	31.90	0.202	0.051
Σ PUFA	13.26	13.29	16.47	0.152	0.012
Saturated/Unsaturated	1.29	1.10	1.08	0.015	0.030
**Atherogenic index** ^**2**^	1.09	0.86	0.77	0.009	0.009

^1^Diets consisted of: Control commercial diet containing sunflower oil; Low-P, low-polyphenols extra-virgin olive oil from *Cima di Bitonto* cultivar; High-P, high-polyphenols extra-virgin olive oil from *Coratina* cultivar; ^2^Calculated according to the equation proposed by Ulbricht and Southgate [30].

The cholesterol is essential for the development of the embryo, and many researchers have tried to decrease the amount of cholesterol in egg yolk due to its risk for cardiovascular disease in humans [11,27]. Azeke and Ekpo [28] suggested that natural polyphenols, catechins, and flavonols have a cholesterol lowering effect on egg by scavenging reactive oxygen species and chelating metal ions. The dietary supplementation of high-polyphenols EVOO may result in a synergistic effect of linoleic acid in decreasing the cholesterol content of egg-yolk. In a recent trial, Zhang and Kim [26] suggested that the mechanism by which olive oil improves the absorption of vitamin A, another fat-soluble vitamin, might facilitate an increase in cholesterol levels in the sera of laying hens.

Table 2 presents the fatty acid compositions of the yolks from eggs of hens submitted to the three treatments. Analysis of fatty acid methyl esters from the egg-yolk lipids of hens revealed they were influenced by the dietary treatments. Egg lipids from hens fed High-P diet showed lower (P < 0.01) values of C14:0, C16:0 than those in control eggs. However, C18:0 did not significantly vary among the three treatments. Hens fed diets with EVOO showed higher (P < 0.01) mean values of C18:1 than those hens fed with control diet. The egg-yolk from high-polyphenols diet showed higher (P < 0.01) levels of fatty acids C18:2 and C18:3 compared to the other dietary groups. A significant decrease of SFA percentage and an increase of the PUFA were observed in eggs from hens fed high-polyphenols EVOO in comparison to the control eggs. In any of the dietary treatments containing EVOO, there was a better saturated to unsaturated fatty acid ratio in comparison with the values obtained for the control eggs. Therefore, it is possible to affirm that all the diets containing EVOO, especially when contained high-polyphenols level, resulted in eggs of higher quality than the control conventional eggs. Even though the SFA/UFA ratio is an important factor from a human nutrition point of view, specific SFA and PUFA have different metabolic effects [29]. Fatty acids can either promote or prevent atherosclerosis and coronary thrombosis, based on their effects on serum cholesterol and low density lipoprotein cholesterol concentrations. For this reason, the atherogenic index have been introduced [30]. The atherogenic index of the egg-yolk lipids differed significantly among dietary treatments, and varied from 1.09, in control diet, to 0.86 and 0.77 in Low-P and High-P diets, respectively (Table 2). The egg-yolks from hens fed high-polyphenols EVOO were characterised by the lowest atherogenic index value, which were related to their lower SFA/UFA ratio. Low values of atherogenic index is recommended for a healthy diet [31]. The C14:0 and C16:0 fatty acids are known to be among the most atherogenic, while C18:0 is thought to be neutral with respect to atherogenicity, but is instead considered to be thrombogenic [32]. Another positive feature arising from the egg-yolk lipid profile in this study was the low or quite similar values for both the atherogenic index, when compared to other meat product [33] or seafood [34].

## Conclusions

In conclusion, after 10 weeks of treatment with each diet type, our results show that EVOO can have high nutritional value. These findings indicate that a supplementation of laying hens with high-polyphenols EVOO reduces egg-yolk cholesterol and enhances the fatty acid profile of yolk lipids, so the use of this oil could have advantages for egg producers as well as advantageous for human nutrition. These findings could have significant consequences for human health and thus deserve further investigation.

## Methods

### Experimental birds and management

This study was conducted in the experimental poultry facility located at the University of Bari ‘Aldo Moro’ , Italy, observing the animal welfare Legislative Decree 116/92, Council Directive 98/58/EC, received in Italy by Legislative Decree 146/2001, and Council Directive 2007/43/CE, received in Italy by the governmental Decree 181/2010 and Legislative Decree 267/2003. A total of 150 ISA Brown laying hens, 18 wk of age, were free-range reared under natural light:dark cycle. The hens were divided into three groups of fifty hens each (two replicates each of 25 birds/group) and were housed in different indoor pens (0.15 m^2^/bird) equipped with feeders and drinkers, with free access to open air runs (3 m^2^/bird).

### Dietary treatments

The diets were based on wheat and soybean meal with added oils at 2.5%. The dietary oils included sunflower oil (Control), extra virgin olive oil from *Cima di Bitonto* variety (low-polyphenols content; Low-P), and extra virgin olive oil from *Coratina* variety (high-polyphenols content; High-P) (Table 3). The extra-virgin olive oils used for experimental diets had a low- and high- total phenol concentration (138 vs. 254 mg/kg) with an different hydroxytyrosol concentrations (17 vs. 43 mg/kg), for *Cima di Bitonto* and *Coratina* variety respectively (Table 4); as determined by Oliveras-López et al. [9]. The sunflower oil used for control diet had very low levels of total polyphenols [35]. The oils were kept in cold room at 4°C prior mixing and the diets were prepared weekly and kept in cold room in air-tight containers. The diets were isocaloric and isonitrogenous containing 17.0% of crude protein (CP) and 2,680 kcal of metabolizable energy/kg of diet, designed to meet or exceed the nutrient requirements for laying hens [36]. The experimental diets were fed to the animals for 10 weeks. Feed and water were provided *ad libitum* throughout the entire trial. Extra-virgin olive or sunflower oils supplementation did not influence feed intake (data not shown). Hens’ mortality was recorded as occurred.

**Table 3:** **Ingredients and chemical analysis of control and experimental diets fed to laying hens**

	**Diet**	
**Ingredients, g/kg as-fed basis**	**Control**	**Experimental**
Corn	541.0	541.0
Soybean meal (48% CP)	175.0	175.0
Calcium carbonate	90.0	90.0
Wheat	75.0	75.0
Alfalfa meal (17% CP)	45.0	45.0
Corn gluten meal (60% CP)	45.0	45.0
Sunflower oil	25.0	--
Extra-virgin olive oil^1^	--	25.0
Dicalcium phosphate	17.0	17.0
Vitamin-mineral premix^2^	2.5	2.5
Sodium chloride	2.5	2.5
Sodium bicarbonate	2.5	2.5
DL-Met	2.0	2.0
L-Lys HCl	2.0	2.0
Yeast	1.5	1.5
Thr	1.0	1.0
**Chemical analysis, %**		
Dry matter	87.54	87.95
Crude protein	16.85	16.71
Crude fibre	3.22	3.47
Crude fat	5.74	5.81
Ash	13.21	13.15
**Calculated analysis**		
ME (kcal/kg of diet)	2,836	2,893
Lys, %	0.86	0.85
Ca, %	4.05	4.08
Met + Cys, %	0.74	0.75
Available P, %	0.36	0.37

Data for chemical analyses are presented as mean values of five observations per treatment.

^1^Experimental diets consisted of: low-polyphenols extra-virgin olive oil from *Cima di Bitonto* cultivar and high-polyphenols extra-virgin olive oil from *Coratina* cultivar.

^2^Provided per kg of product: 2,500.000 IU vitamin A; 300,000 IU vitamin D_3_; 7,500 mcg 25-hydroxycholecalciferol; 6,000 mg vitamin E; 500 mg vitamin K_3_; 4,000 mg vitamin PP; 300 mg vitamin B_1_; 1,000 mg vitamin B_2_; 2,000 mg D-pantothenic acid; 400 mg vitamin B_6_; 3 mg vitamin B_12_; 150 mg folic acid; 20 mg D-biotin; 10,000 mg Fe; 1,000 mg Cu; 30,000 mg Mn; 40 mg Co; 15,000 mg Zn; 200 mg I; 20 mg Se.

**Table 4:** **Polyphenol profile (ppm, mg/kg) and fatty acid composition (% on total) of the low-polyphenols** ***Cima di Bitonto*** **and high-polyphenols** ***Coratina*** **extra-virgin olive oils**

**Determined by HPLC** ^**1**^	**Extra-virgin olive oil**		
	***Cima di Bitonto***	***Coratina***	**SEM**
**Phenol composition**			
Hydroxytyrosol	16.92	42.98	0.052
Tyrosol	16.98	66.25	0.045
3,4-DHPEA-EDA	32.63	39.44	0.021
*p*-HPEA-EDA	7.93	38.42	0.016
(+)-1-Acetoxypinoresinol	11.44	24.16	0.014
(+)-Pinoresinol	2.47	13.34	0.009
Luteolin	4.16	5.25	0.005
3,4-DHPEA-EA	1.77	14.64	0.019
Apigenin	3.82	4.51	0.003
*p*-HPEA-EA	2.29	4.59	0.002
**Total phenols**	137.85	253.58	0.521
**Fatty acid composition**			
C16:0 Palmitic	18.75	14.77	0.201
C18:1 Oleic	72.71	77.07	0.147
C18:2 Linoleic	7.79	7.04	0.198
C18:3 Linolenic	0.75	1.12	0.075

^1^Each value represents the mean of five replicate analyses; SEM, standard error of the means.

### Sample collection and procedures

Samples of diets were ground in a hammer mill with a 1 mm screen and analysed in triplicate for dry matter (DM, 945.15), ash (967.05), crude protein (CP, Kjeldahl N × 6.25, 990.03), crude fiber (978.10) and ether extract (945.16) according to AOAC International [37].

Eggs were collected daily and egg production was calculated on a hen-day basis. Eggs with any adhering manure were classed as dirty, and the percentage calculated. Eggs produced the last day of each week on trial were individually weighed. Egg mass production per hen per day was calculated as laying percentage multiplied by average egg weight of the hen. Feed conversion ratio (FCR) was calculated as gram feed consumption per day per hen divided by gram egg mass per day per hen Eggs were analyzed for their interior and exterior quality as reported by Laudadio and Tufarelli [30]. Eggs were examined for shell quality by specific gravity. Shell thickness (with shell membrane) of the eggs (10% of the daily egg produced) was measured by micrometer. Shell thickness was a mean value of measurements at 3 locations on the eggs (air cell, equator, and sharp end). Breaking strength of uncracked eggs was determined with a testing machine (model 1140, Instron Ltd., Bucks, UK). Egg components (as albumen, yolk and shell) were measured by weekly breakouts on two eggs per replicate pen and expressed as percentage of egg weight. The Haugh unit was calculates as: Haugh units (%) = 100 × log (H + 7.57 − 1.7 W^0.37^, where H is the height of the albumen and W is the weight of the egg) according the formula proposed by Card and Nesheim [38]. Egg yolk color was scored using the 15-point scale (colour scale from 15, dark orange to 1, light pale) of the DSM yolk color fan (DSM Nutritional Products Ltd., Basel, Switzerland). Blood samples (2.0 ml) from each individual layer were collected weekly during the whole feeding period. Blood was collected from the brachial wing vein using sterilized syringes and needles. After 1 h standing at room temperature, serum was isolated by centrifugation at 1,150 × *g* for 10 min. Serum samples were stored at −80°C until further analysis.

### Yolk and serum cholesterol contents

The yolk cholesterol concentrations were determined sampling egg yolks (1 g) weekly saponified with 20 ml of 33% ethanolic KOH in tightly-capped tubes placed in a 60°C water bath for 1 h. The mixture was then cooled in ice water, and 5 ml of distilled water was added. Cholesterol in unsaponifiable fractions was extracted twice with 5 ml of hexane. The resulting aliquot of hexane containing cholesterol was dried under nitrogen, redissolved in 5 ml of hexane, and injected into a gas chromatograph (HP-6890 N, Palo Alto, CA, USA). 5 α-cholestane (Sigma–Aldrich) was used as an internal standard. A split inlet (split ratio, 100:1) was used to inject samples into a capillary column (HP-5, Agilent, Steven, CA, USA; 30 m × 0.53 mm × 0.5 μm), and the ramped oven temperature was 270°C isothermal, detector temperature was 300°C, and inlet temperature was 210°C. The N_2_ served as the carrier gas at a constant flow rate of 1.0 ml/min. Total cholesterol concentrations in the plasma was analysed independently by UV spectrophotometer using commercial kits (Diasys, Diagnostic systems, Germany).

### Polyphenols and fatty acid analyses

Quantitative and qualitative analysis of phenolic fraction of the extra virgin olive oils was performed according to the COI/T20/29doc (International Olive Council) for olive oil. The method is based on direct extraction of the phenolic minor polar compounds from olive oil by means of a methanol solution and subsequent quantification by high-performance ternary gradient liquid chromatograph (HPLC) [5]. After direct extraction of the phenolic minor polar compounds by means of a methanol solution, an aliquot of the supernatant phase was taken and filtered through a 0.45-mm PVDF filter, injected into the HPLC system equipped with C18 reverse-phase column (4.6 mm × 25 cm), type Spherisorb ODS-2 (5 mm), 100 A°, with spectrophotometric UV detector at 280 nm and integrator. The content of the biophenols was expressed in milligrams of tyrosol per kilogram of oil and was calculated by measuring the sum of the areas of the related chromatographic peaks. The composition of the isolated phenolic fraction is detailed in Table 4.

In preparation for fatty acid (FA) composition analysis, samples of oil and egg-yolk (5 g each) were freeze-dried. Briefly, methyl heptadecanoate (no. 51633, Fluka, St. Louis, MO) was dissolved into n-hexane (1 mg/mL) as an internal standard. Methyl esters of the FA were prepared [39]; samples (300 mg each) and 5 mL of internal standard were incubated (2 h at 80°C) with methanolic acetyl chloride in a total volume of 9 mL. After cooling to room temperature, 7 mL of 7% (wt/vol) K_2_CO_3_ was added with mixing, and then the organic phase was collected after centrifuging at 1,500 × *g* for 2 min at 4°C. The FA methyl esters were fractionated over a CP-SIL883 column (100 m × 0.25 mm i.d., film thickness 0.20-μm fused silica; Varian, Palo Alto, CA) in a Shimadzu (model 2GC17A, Shimadzu, Kyoto, Japan) gas chromatograph with a Hewlett-Packard HP 6890 gas system (Palo Alto, CA) and using flame ionization detection. Helium was used as the carrier gas at a constant flow rate of 1.7 mL/min. The oven temperature was programmed as follows: 175°C, held for 4 min; 175 to 250°C at 3°C/min; and then maintained for 20 min. The injector port and detector temperature was 250°C. Samples (1 μL) were injected by an auto-sampler. Output signals were identified and quantified from the retention times and peak areas of known calibration standards. Composition was expressed as percentages of the total FA. The atherogenic index was calculated according to the equation proposed by Ulbricht and Southgate [30].

### Statistical analysis

Data were analyzed using the one-way ANOVA option of the GLM of SAS/STAT software [40] as a completely randomized design with the dietary treatments or oil sources as main effects. The statistical model used was: Y_ijk_ = μ + P_i_ + R_ij_ + ε_ijk_, where Y_ijk_ = response variables from each individual replication or pen, μ = the overall mean; P_i_ = the effect of dietary oil source; R_ij_ = the inter-experimental unit (replications) error term; and ε_ijk_ = the intra-experimental unit error term. When there was a significant *F*-value, means were compared by the Student-Newman-Keul’s method. Significance implies P < 0.05 unless stated otherwise.
